# Ecological theatre and the evolutionary game: how environmental and demographic factors determine payoffs in evolutionary games

**DOI:** 10.1007/s00285-012-0573-2

**Published:** 2012-08-31

**Authors:** K. Argasinski, M. Broom

**Affiliations:** 1 Department of Mathematics, University of Sussex, Brighton, BN1 9QH UK; 2 Centre for Mathematical Science, City University London, Northampton Square, London, EC1V 0HB UK

**Keywords:** Replicator dynamics, Mortality, Fertility, Eco-evolutionary feedback, Trade-off, Density dependence, 92D40

## Abstract

In the standard approach to evolutionary games and replicator dynamics, differences in fitness can be interpreted as an excess from the mean Malthusian growth rate in the population. In the underlying reasoning, related to an analysis of “costs” and “benefits”, there is a silent assumption that fitness can be described in some type of units. However, in most cases these units of measure are not explicitly specified. Then the question arises: are these theories testable? How can we measure “benefit” or “cost”? A natural language, useful for describing and justifying comparisons of strategic “cost” versus “benefits”, is the terminology of demography, because the basic events that shape the outcome of natural selection are births and deaths. In this paper, we present the consequences of an explicit analysis of births and deaths in an evolutionary game theoretic framework. We will investigate different types of mortality pressures, their combinations and the possibility of trade-offs between mortality and fertility. We will show that within this new approach it is possible to model how strictly ecological factors such as density dependence and additive background fitness, which seem neutral in classical theory, can affect the outcomes of the game. We consider the example of the Hawk–Dove game, and show that when reformulated in terms of our new approach new details and new biological predictions are produced.

## Introduction

The relationship between ecology and evolution is one of the major topics under current development in biology. This research perspective was formulated by Evelyn Huthinson in his essay “Ecological Theatre and the Evolutionary Play” (Hutchinson 1965) describing the necessity of the investigation of ecological factors that shape natural selection. This idea was very influential, and the topic of feedback between ecology and evolution originated by this work is still discussed (Post and Palkovacs 2009). The basic methodology applied to the modeling of ecological and evolutionary problems is that of population dynamics. As postulated by Geritz and Kisdi (2011), realistic mathematical models should be mechanistic and derived from basic principles describing individual behaviour. A natural methodology which satisfies this assumption is evolutionary game theory. Methods of evolutionary game theory (Maynard Smith 1982; Hofbauer and Sigmund 1988, 1998; Cressman 1992; Weibull 1995) are very popular in many fields not related to biology, such as social sciences, economics and even telecommunication engineering. However, in the field of evolutionary ecology modern developments in evolutionary games are somewhat marginalized. Criticism of mathematical methods arise from the individual based approach (Uchmański and Grimm 1986) presented by Adam Lomnicki in the first book in the field, “Population ecology of individuals” (Lomnicki 1988). This approach inspired the development of computer simulation techniques based on multiagent systems (Grimm and Railsback 2005. One of the most serious complaints about evolutionary games is that they lack ecological details and that game theoretic models are too abstract and idealized (as was convincingly highlighted by Mylius 1999 by example of the Battle of the Sexes game). This problem can be shown by the example of the extensively used Hawk–Dove game, described by the payoff matrix:

$$\begin{aligned} T=\left( \begin{array}{c|cc}&H&D \\ \hline H&0.5(G-C)&G \\ D&0&0.5G \end{array} \right) \end{aligned}$$

where $G$ means the “benefit” in fitness of obtaining the reward and $C$ the additive “cost” of conflict. The problem is as follows. In the standard approach to evolutionary games and replicator dynamics, differences in fitness can be interpreted as an excess from the mean Malthusian growth rate in the population (Roff 2008). In the underlying reasoning, related to an analysis of “costs” and “benefits”, there is the implicit assumption that fitness can be described in some kind of units. However, in most cases these units of measure are not explicitly specified. Then the question arises: are these theories testable? How can we measure “benefit” or “cost”? In the case of the above matrix it is possible that the pure benefit $G$ can be estimated from observations of outcomes of interactions between Doves. However, imagine some generalization of this basic model, where Doves’ reproductive success is influenced by a different cost caused by for example females mating preferences for Hawks. In this case there are no strategies that obtain the pure benefit $G$. When “benefit” is an expected increase in reproductive success and “cost” is a decrease in the same value, how can we empirically measure the number of offspring that were never raised? Models should ideally be formulated in parameters that can be easily measured in nature. A good alternative to this abstract additive cost/benefit analysis is the language of classical demography that is strongly bonded with reality. Also the application of demographic notions in game theoretic models can build a bridge between evolutionary game theory and life history theory where demographic tools are widely applied (Roff 1993; Stearns 1992). In the terminology of demography payoffs in evolutionary games can be described in two types of currency: mortality and fertility. Therefore instead of the above payoff matrix, we shall consider these two components separately, summarized by two matrices of payoffs. The survival or mortality matrix,

$$\begin{aligned} S=\left( \begin{array}{c|cc}&H&D \\ \hline H&s&1 \\ D&1&1 \end{array} \right) \end{aligned}$$

where $s<1$ is the survival probability of a fight between Hawks, and the fertility matrix containing the expected number of newborns produced in effect from the interaction,

$$\begin{aligned} F=\left( \begin{array}{c@{\quad }c@{\quad }c}&H&D \\ H&0.5W&W \\ D&0&0.5W \end{array} \right). \end{aligned}$$

How can these matrices be combined into the individual’s payoff function? This is the goal of this paper. To find an answer we should investigate how population dynamics induced by these demographic parameters determine the selection of strategies.

## The basic assumptions of classical theory

When a population with a finite number of individual strategies is considered, then a system of differential equations called *replicator dynamics* can be defined. It describes changes of population state in time and can be derived in the following way. Assume that we have a finite number $I$ arbitrary chosen strategies. For each strategy some payoff function $r_{i}$ is assigned (for example matrix form, as in the classical Hawk–Dove game, however the form of payoff function depends on the modelled problem and can be more complicated). Then the growth of the population of $i$-strategists can be described by the Malthusian equation

1 $$\begin{aligned} \dot{n}_{i}=n_{i}r_{i}. \end{aligned}$$

Then by following a change of coordinates,

2 $$\begin{aligned} q_{i}=\dfrac{n_{i}}{n} \end{aligned}$$

where $n=\sum _{i=1}^{I}n_{i}$ is the population size, we can derive a system of ordinary differential equations (see, e.g. Cressman 1992),

3 $$\begin{aligned} \dot{q}_{i}=q_{i}(r_{i}-\bar{r}) \end{aligned}$$

where $\bar{r}=\sum _{i=1}^{I}q_{i}r_{i}$ is the average payoff in the population. It has been shown that the replicator dynamics for every complicated multipopulation and density dependent evolutionary game can be reduced to the consideration of the replicator dynamics of the relative frequencies within a single population together with a single equation for the population size dynamics (Argasinski 2006). Therefore the equation

4 $$\begin{aligned} \dot{n}=n\bar{r} \end{aligned}$$

together with (3) describes the evolution of the population. Thus the values of the functions $r_{i}$ should be interpreted as Malthusian parameters and their arguments should be strategy frequencies $q$ and population size $n$ (although we can imagine more complicated models, therefore arguments of payoffs will be not explicitly specified in the formulas). The importance of the turnover of individuals for selection dynamics in density dependent models has also been shown (Argasinski and Kozłowski 2008). To induce turnover, some neutral mortality (the same for all strategies while payoffs are interpreted as per capita number of newborns) on adult individuals should be explicitly considered in the model. However, selection is driven by differences in fertility. It is obvious that in some cases survival of parental individuals may be a reward in the game. In this paper we will generalize this result to the cases where adult mortality may differ for different strategies. Therefore instead of the Malthusian function $r_{i}$, we shall consider the fertility payoff $W_{i}$, the per capita number of offspring of the $i$-th type, and the mortality payoff $d_{i}$ (or survival $s_{i}=1-d_{i}$), the probability of death of a single adult individual of the $i$-th type for each strategy.

## Models of fertility and mortality

As described above, our model will consist of the parameters $n_{i}$ and functions $W_{i}$ and $d_{i}$. This is a natural language for discrete systems while the continuous approach uses for example the mortality rate instead of the probability of death. However this meaning is important for our mechanistic interpretation and empirical tests of predictions of developed models. A rate parameter describes change with respect to time while the probability of death is a parameter characterizing a single interaction, therefore the mortality rate should be a function of the probability of death during a single event and the number of these events in a unit of time. This interpretation is natural for discrete systems, whereas continuous models are described by phenomenological rates of change during an infinitesimal time-step. This derivation method, natural from the point of view of classical mechanics can be problematic to the mechanistic interpretation in population dynamics models. The problem is that below some critical threshold there are no significant changes of the population state and the model falls into Markovian processes described by intensities of events which are parameters which are difficult to measure. This makes empirical testing and falsification of the theory difficult. Fortunately, changes in the population size can be approximated using Taylor series operating on a non-infinitesimal time interval when the trajectory is nearly linear. In effect model parameters are described by the average outcome of an ensemble of events realised during that interval described by the number of newborns and the fraction of dead individuals. In that case there is no need to use the word probability, while the model can be described by empirically measurable fractions. In effect the mechanistic interpretation from discrete models can be transferred to the continuous case too (the equivalence of discrete and continuous models for values of fitness near 1 was mentioned by Hartl and Clark 2006). This is justified in Appendix 1 where the similarities and differences between the discrete and continuous cases are analyzed, and we see that we can write our basic population growth equation as

5 $$\begin{aligned} \dot{n}_{i}=n_{i}W_{i}-n_{i}d_{i}=n_{i}(W_{i}-d_{i}). \end{aligned}$$

Where $W_i$ is the number of newborns and $d_i$ is the fraction of dead individuals. In the following we introduce a modelling framework based upon four factors, fertility and three distinct types of mortality. We shall build up to our model with a number of applied frameworks, introducing new factors at each stage.

### Framework I: fertility and post-reproduction mortality

Here we will extend the replicator dynamics to the case where mortality and fertility can be explicitly considered, not only the Malthusian parameter.

The basic population growth equation in this case will be given by Eq. (5). We can then derive the dynamics on the related frequencies in the same way as in classical replicator dynamics. In effect we obtain the following variant of the replicator equations that can be called the *sex and violence equations*,

6 $$\begin{aligned} \dot{q}_{i}=q_{i}(W_{i}-\bar{W}-d_{i}+\bar{d})=q_{i}((W_{i}-\bar{W})-(d_{i}- \bar{d})) \end{aligned}$$

where $\bar{W}=\sum q_{i}W_{i}$ and $\bar{d}=\sum q_{i}d_{i}$ are averages over the population (as in the standard replicator dynamics). Similar equations are known and applied in population genetics (see Hofbauer and Sigmund 1988, 1998; Cressman 1992). However, in the field of evolutionary games this type of equation has not been sufficiently appreciated and rigorously analyzed.

Alternatively to using the probability of death, we can use the survival probability given by $s_{i}=1-d_{i}$. We may wish to talk in terms of either of these factors, but for convenience from now on we will use survival probabilities in our formulae (except for the latter part of the section on the Hawk–Dove example game). Equation (6) thus becomes

7 $$\begin{aligned} \dot{q}_{i}=q_{i}(W_{i}-\bar{W})-(1-s_{i}-1+ \bar{s})=q_{i}(W_{i}-\bar{W} )+(s_{i}-\bar{s}), \end{aligned}$$

where $$\bar{s}=\sum _{i}q_{i}s_{i}.$$

Therefore, in this case selection is driven by the interplay between the fertility and mortality stages (both described by terms in brackets). In the case where mortality or fertility are selectively neutral (i.e. the same for all strategies, for example when the game affects mortality and there are no increments in fertility) one factor vanishes and we obtain the standard replicator dynamics. Since payoffs $W_{i}$ and $s_{i}$ may be density dependent, the above system should be completed by an equation on the population size $n=n_{1}+\cdots +n_{I}$ (Argasinski 2006):

8 $$\begin{aligned} \dot{n}&= n\sum \limits _{i}q_{i}(W_{i}+s_{i}-1)\end{aligned}$$

9 $$\begin{aligned} \dot{n}&= n\left(\sum \limits _{i}q_{i}W_{i}+\sum \limits _{i}q_{i}s_{i}-1\right) =n( \bar{W}+\bar{s}-1). \end{aligned}$$

### Framework II: fertility and pre-reproduction mortality with mortality-fertility frequency dependent trade-offs

It is clear that in continuous models the probability of two independent events occurring within a given time interval tends to zero as the length of the time interval tends to zero. However, we should remember that biological reality can be very complicated and the outcomes of a single event can be very complex. For example, consider a male involved in a mating conflict where victory will lead to an immediate mating opportunity, i.e. there is a chain of conditional stages caused by a single interaction event. He may be killed instantly during a fight, or survive then mate successfully and die afterwards due to infection of his wounds. Death has occurred in both cases, but in the second case mating has also occurred, and it is important to distinguish the two different types of mortality. A similar idea has been known in population genetics for a long time (Prout 1965).

Framework I is a good model when mortality pressure acts after reproduction, i.e. individuals reproduce first and are then eliminated, so mortality does not affect fertility. However, the idea of the trade-off that is the cornerstone of life history models states that individuals should die or reproduce, but cannot do both of these things at the same time. There are situations when conflicts occur between individuals before mating and mortality acts before reproduction. Note that in Eq. (5) $n_{i}$ describes the initial number of individuals prior to deaths caused by mortality pressure. When mortality is acting before reproduction then only $(1-d_{i})n_{i}=s_{i}n_{i}$ will reproduce. This leads to a modification of the above formalism that can be called the *sex or violence equations*, and here we approach unknown ground. Let us start from equations on the sizes of the subpopulations of the different types, where (5) is replaced by

10 $$\begin{aligned} \dot{n}_{i}=s_{i}n_{i}W_{i}-n_{i}(1-s_{i})=n_{i}( s_{i}W_{i}+s_{i}-1) . \end{aligned}$$

However, this form is relevant only when $s_{i}$ and $W_{i}$ are constants or frequency independent functions. If $s_{i}(q)$ and $W_{i}(q)$ (where $q$ is the vector of relative frequencies of strategies) are frequency dependent functions, then they are the mean payoffs of $i$-strategists averaged over interactions with other strategists. Therefore the term $s_{i}W_{i}$ should be an average over the product of mortality and fertility payoffs against a random opponent strategy, rather than just a multiplication of average values. To fix this problem we introduce a frequency dependent mortality-fertility trade-off function

11 $$\begin{aligned} V_{i}(q,s_{i},W_{i})=\sum \limits _{j}q_{j}s_{i}(e_{j})W_{i}(e_{j}) \end{aligned}$$

(where $$e_{j}$$ is the unit vector with 1 in the $$j$$-th position), later for simplicity denoted $$V_{i}$$ [note that in the specific case where $$s_{i}$$ and $$W_{i}$$ are not frequency dependent, then (11) collapses to $$ V_{i}=s_{i}W_{i}$$]. Therefore in the general case (10) is replaced by

12 $$\begin{aligned} \dot{n}_{i}=n_{i}V_{i}-n_{i}(1-s_{i})=n_{i}( V_{i}+s_{i}-1). \end{aligned}$$

Dividing by $n$ as in Eq. (2), and using (7), we obtain the frequency equations

13 $$\begin{aligned} \dot{q}_{i}=q_{i}\left(\left(V_{i}- \sum _{j}q_{j}V_{j}\right)+\left(s_{i}-\bar{s}\right)\right). \end{aligned}$$

The difference between (7) and (13) is that $W_{i}$ is replaced by $V_{i}$. Then the equation on the population size will be;

14 $$\begin{aligned} \dot{n}=n\sum \limits _{i}q_{i}(V_{i}+s_{i}-1) =n( \bar{V}+\bar{s} -1) \end{aligned}$$

which is Eq. (9) with $\bar{V}$ replacing $\bar{W}$, where $\bar{V}=\sum _{i}q_{i}V_{i}$.

### Framework III: combining different mortality pressures

Imagine now that there are two separate mortality pressures on our population. One mortality pressure described by $d_{i}=1-s_{i}$ affects fertility by excluding dead individuals from reproduction as described in Framework II, the second mortality pressure, described by $m_{i}=1-b_{i}$, only removes some fraction of adult individuals from the population after reproduction, as described earlier in Framework I, where $m_{i}$ is the probability of death after reproduction and so $b_{i}$ is the equivalent probability of survival. As before we will only include $b_{i}$ in our formulae for the sake of convenience. Let us start from the equation on the number of individuals, where (5) and (12) are now special cases of

15 $$\begin{aligned} \dot{n}_{i}=n_{i}V_{i}-n_{i}(1-s_{i})-n_{i}(1-b_{i})s_{i}=n_{i}( V_{i}-1+b_{i}s_{i}). \end{aligned}$$

Again, by dividing by $n$ we obtain frequency equations

16 $$\begin{aligned} \dot{q}_{i}=q_{i}\left(\left(V_{i}-\sum \limits _{j}q_{j}V_{j}\right) +\left(s_{i}b_{i}-\sum \limits _{i}q_{i}s_{i}b_{i}\right)\right). \end{aligned}$$

The equation on the population size has the form

17 $$\begin{aligned} \dot{n}=\sum \limits _{i}n_{i}(s_{i}(W_{i}+b_{i})-1) =n\sum \limits _{i}q_{i}(s_{i}(W_{i}+b_{i})-1)=n(\bar{V}+\bar{B}-1) \qquad \end{aligned}$$

where $\bar{V}$ is as in Eq. (14) but $\bar{s}$ is replaced by $\bar{B}=\sum _{i}q_{i}s_{i}b_{i}$. Note that we can imagine more complex cases combining many mortality sources of both types.

### Framework IV: adding neutral density dependence

Populations cannot grow to infinity, therefore there should always be some density dependence. We can extend the model to include density dependence by adding offspring mortality described by the logistic suppression coefficient $(1-n/K)$, where we multiply all fertility parameters $W_{i}$ by this coefficient. Such logistic suppression is used in population genetics (Hofbauer and Sigmund 1988, 1998), and in game theoretic models of the ideal free distribution (Cressman et al. 2004; Cressman and Krivan 2006, 2010). However, in the classical phenomenological form it may produce paradoxical and unrealistic predictions (Geritz and Kisdi 2011). Our approach to logistic growth was originated in Kozłowski (1982). Logistic suppression can be interpreted as the per capita mortality of juvenile individuals, which is selectively neutral, i.e. the same for each strategy. It was shown that without an additional mortality pressure on adult individuals that induces turnover of individuals, selection stops when the population reaches carrying capacity. However by the addition of some background neutral mortality of adult individuals this suppression of selection is avoided (Argasinski and Kozłowski 2008). We note that carrying capacity can be interpreted as a number of habitats rather that a population equilibrium. Therefore, under the assumption that individuals cannot live without a habitat, it makes no sense to consider an initial population size greater than the carrying capacity, and we shall assume that population size cannot exceed the carrying capacity.

The approach developed in this paragraph is a generalization of results from (Argasinski and Kozłowski 2008) to the case when additive adult mortality pressure can differ for different strategies. Thus the system (16) and (17) will become

18 $$\begin{aligned} \dot{q}_{i}&= q_{i}\left(1-\frac{n}{K}\right) \left( \left(V_{i}-\sum \limits _{j}q_{j}V_{j}\right)+\left(s_{i}b_{i} -\sum \limits _{j}q_{j}s_{j}b_{j}\right)\right),\end{aligned}$$

19 $$\begin{aligned} \dot{n}&= n\left( \bar{V}\left( 1-\frac{n}{K}\right) +\bar{B}-1\right). \end{aligned}$$

This is the most complex case of our framework; mortality pressure can be easily introduced to the simpler cases analyzed before by setting $s_{i}$ or $b_{i}$ equal to 1. We have introduced a number of terms in the paper so far. A summary of the important components of our framework is given in Table 1.

**Table 1:** Important symbols for our modelling framework

Symbols	Meaning
$B$	The benefit in the classical Hawk–Dove game
$C$	The cost in the classical Hawk–Dove game
$T$	The classical Hawk–Dove game payoff matrix
$S$	The survival (mortality) matrix
$F$	The fertility matrix
$n_{i}$	The number of individuals of the $i$-th type
$r_{i}$	Function describing the Malthusian parameter of the $i$-th type
$n$	The total population size
$K$	The population carrying capacity
$q_{i}=n_{i}/n$	The relative frequency of the $i$-th type
$W_{i}$	Reproductive success function of the $i$-th type
$s_{i}=1-d_{i}$	The pre-reproduction survival probability function of the $i$-th type
$b_{i}=1-m_{i}$	The post-reproduction survival probability function of the $i$-th type
$V_{i}(q,s_{i},W_{i})$	The frequency dependent mortality-fertility tradeoff of the $i$-th type
$W_{b}$	The background fertility

The stationary population sizes of Eq. (19) are $\tilde{n}=0$ and

20 $$\begin{aligned} \bar{V}\left(1-\frac{n}{K}\right) +\bar{B}-1=0. \end{aligned}$$

Equation (20) satisfies

21 $$\begin{aligned}&1-\frac{\check{n}}{K}=\frac{1-\bar{B}}{\bar{V}}\Rightarrow \end{aligned}$$

22 $$\begin{aligned}&\check{n}(q)=K\left( 1-\frac{1-\bar{B}}{\bar{V}}\right)=K\left( 1-\frac{1-\sum _{i}q_{i}s_{i}b_{i}}{\sum _{i}q_{i}V_{i}}\right). \end{aligned}$$

The stability of (22) comes directly from the negative linearity in $n$ of the expression from Eq. (19). Note that (22) describes an attractor manifold of the population size dynamics, conditional on the current population state described by the vector of strategy frequencies. For a population not to be become extinct we need $\check{n}>0$ and so $1-\bar{B}<\bar{V}$, which means that the per capita death rate of adult individuals should be smaller than the per capita reproductive success. For a zero death rate we obtain $\check{n}=K$. We note Eq. (19) behaves properly in the sense that the trajectory of the dynamics will not leave the interval $[0,K]$.

### The system in ecological equilibrium

We can imagine situations when we are interested in modelling only frequency dependence. In some papers only frequency dynamics is considered under the assumption that the population is in balance between mortality and fecundity, so that we can “forget” about population size. In addition, in many papers this is realized by the assumption of “weak selection”, which means that most birth and death events are independent of strategy. This is problematic in, for example, mating conflicts where the majority of births and deaths are affected by strategies, and the chance to mate without competition with other candidates can be quite low. Therefore, the ecological dynamics can be seriously affected by outcomes of the evolutionary game. However, instead of forgetting about density or separating it from the game dynamics, it is biologically more realistic to assume that the population size is determined by the balance between mortality and fecundity (Argasinski and Kozłowski 2008). Therefore the phase space of the system is reduced to a stable manifold described by the stationary points of the size equation, where the population size traces changes of population composition (an assumption used in adaptive dynamics Vincent and Brown 2005; Dercole and Rinaldi 2008). This approach is similar to the concept of the stationary density surface (Cressman and Garay 2003). The difference is that here we do not assume separation of timescales between size and frequency dynamics. Substituting $\check{n}$ into the logistic suppression coefficient and using (22) we obtain the frequency dependent multiplicative modifier

23 $$\begin{aligned} 1-\frac{n}{K}=\frac{1-\sum _{i}q_{i}s_{i}b_{i}}{\sum _{i}q_{i}V_{i}}. \end{aligned}$$

Then (18) becomes

24 $$\begin{aligned} \dot{q}_{i}=q_{i}\left( \left( \dfrac{1-\sum _{j}q_{j}s_{j}b_{j}}{ \sum _{j}q_{j}V_{j}}\right) \left(V_{i}-\sum \limits _{j}q_{j}V_{j}\right)+ \left(s_{i}b_{i}-\sum \limits _{j}q_{j}s_{j}b_{i}\right)\right) \end{aligned}$$

which together with (22) represents the whole system.

Then from the mathematical point of view the system is simplified because, instead of a system of two differential equations, we obtain a single differential equation (24) and the function of the population state $\check{n}$ (22), describing the population size. On the other hand the equation on frequency dynamics is more complicated and behaviour may be more complex. There is a unique restpoint for the density independent case which now become a function of $n$, because then the fertility bracket is multiplied by formula (23). Thus, stationary states should be described by stable frequencies and densities.

### Is background fitness really background?

In the classical approach to evolutionary games and replicator dynamics some additive constant that vanishes in the replicator equation is called the background fitness. Following the distinction between mortality and fertility this concept splits into background mortalities of both types that can be modelled by selectively neutral $d=1-s$ and $m=1-b$ and background fertility $W_{b}$ that can be added for each $W_{i}.$ In the new approach only pre-reproduction mortality is really neutral, because it appears as a multiplicative factor of the whole right side of equations, affecting the rate of convergence. The background mortality $1-b$ reduces the influence of the mortality stage, because it appears as a multiplicative factor of the bracket describing this stage $b(s_{i}-\sum _{i}q_{i}s_{i})$ (see Eq. 18). The introduction of background fertility causes reinforcement of the mortality stage by increasing the multiplicative weight of the bracket containing the dynamics of this stage. By adding $W_{b}$ to all of the fertility values $W_{i}$, we obtain

25 $$\begin{aligned} \breve{V}_{i}=\sum \limits _{j}q_{j}s_{i}(e_{j})W_{b}+ \sum \limits _{j}q_{j}s_{i}(e_{j})W_{i}(e_{j})=W_{b}s_{i}+V_{i}. \end{aligned}$$

In effect (18) and (19) become

26 $$\begin{aligned} \dot{q}_{i}&= q_{i}\left( \left( 1-\frac{n}{K}\right) \left(V_{i}-\sum \limits _{j}q_{j}V_{j}\right)+\left( 1-\frac{n}{K}\right) W_{b}\left(s_{i}-\sum \limits _{j}q_{j}s_{j}\right)\right.\nonumber \\&\left.+\left(s_{i}b_{i}-\sum \limits _{j}q_{j}s_{j}b_{j}\right)\right),\end{aligned}$$

27 $$\begin{aligned} \dot{n}&= n\left( \left( 1-\frac{n}{K}\right) \left( W_{b}\sum _{i}q_{i}s_{i}+\sum _{i}q_{i}V_{i}\right) +\sum _{i}q_{i}s_{i}b_{i}-1\right), \end{aligned}$$

where the equilibrium population size from (22) is

28 $$\begin{aligned} \check{n}=K\left( 1-\dfrac{1-\sum _{i}q_{i}s_{i}b_{i}}{W_{b} \sum _{i}q_{i}s_{i}+\sum _{i}q_{i}V_{i}}\right). \end{aligned}$$

A second effect is an increase in the value of the equilibrium population size and in effect greater suppression of the fertility stage (a lower value of the logistic coefficient); exclusion creates an additional penalty for dead individuals because they lose their whole reproductive success $W_{b}+W_{i}$.

## An application: a Hawk–Dove example game

We can apply our model to an example game, a version of the Hawk–Dove game of Maynard Smith. The game is summarised by the following four parameters: $W\in (0,\infty )$ is the expected number of offspring produced after winning an interaction, $W_{b}\in [0,\infty )$ is the background fertility (the number of offspring that can be obtained without fighting), $s\in (0,1)$ is the survival probability of fighting (affecting fertility), and $b$ is the basic survival probability due to senescence, seasonal mortality etc. (not affecting fertility). Then the survival matrix is now $bS$, and the fertility matrix is $F$, where $S$ and $F$ are as defined in the Sect. 1. We shall write $F=WP$, so that the matrix $P$ is

$$\begin{aligned} P=\left( \begin{array}{c|cc}&H&D \\ \hline H&0.5&1 \\ D&0&0.5 \end{array} \right). \end{aligned}$$

Then to apply the methods described in the previous sections we use the following expressions: $s_{i}=(Sq^{T})_{i}=e_{i}Sq^{T}, \sum q_{i}s_{i}=qSq^{T}, V_{i}=(S\cdot Pq^{T})_{i}=e_{i}(S\cdot P)q^{T}$, and $\sum q_{i}V_{i}=q(S\cdot P)q^{T}.$

Given that there are only two strategies, $q_{d}=1-q_{h}$ so that we only need to evaluate the population size and the fraction of Hawks at any time. Using $e_{1}$ as the vector $(1,0)$ we obtain the following equations from (26) and (27)

29 $$\begin{aligned} \dot{q}_{h}&= q_{h}\left( \left( 1-\dfrac{n}{K}\right) \left( W_{b}\left( e_{1}Sq^{T}-qSq^{T}\right) +W\left( e_{1}S\cdot Pq^{T}-qS\cdot Pq^{T}\right) \right)\right.\nonumber \\&\left.+\,b(e_{1}Sq^{T}-qSq^{T})\right) \end{aligned}$$

and

30 $$\begin{aligned} \dot{n}=n\left( \left( qSq^{T}W_{b}+qS\cdot Pq^{T}W\right) \left( 1-\frac{n}{K}\right) +qSq^{T}b-1\right). \end{aligned}$$

After calculations and auxiliary substitution $d=1-s$ (see Appendix 2 for details) we obtain:

31 $$\begin{aligned} \dot{q}_{h}&= q_{h}(1-q_{h}) \left( \left( 1-\dfrac{n}{K}\right) (0.5W( 1-q_{h}d) -W_{b}q_{h}d)-bq_{h}d\right),\end{aligned}$$

32 $$\begin{aligned} \dot{n}&= n\left((1-q_{h}^{2}d) \left( \left( W_{b}+0.5W\right) \left( 1-\frac{n}{K}\right) +b\right) -1\right) . \end{aligned}$$

Two rest points of this system are $q_{h}=0$ and $1$. A nontrivial rest point, which becomes the attractor manifold for the density dependent case, (for detailed calculation see Appendix 3) is given by

33 $$\begin{aligned} \tilde{q}_{h}(n)=\dfrac{0.5W\left( 1-\dfrac{n}{K}\right) }{d\left( \left(W_{b}+0.5W\right) \left( 1-\dfrac{n}{K}\right) +b\right)}. \end{aligned}$$

We find a stable population size if either $\tilde{n}=0$ or the following positive restpoint which is conditional on the actual Hawk strategy frequency (describing the attractor manifold parametrized by $q_{h}$)

34 $$\begin{aligned} \tilde{n}(q_{h})=K\left( 1-\dfrac{1-b(1-q_{h}^{2}d) }{(W_{b}+0.5W) (1-q_{h}^{2}d) }\right) . \end{aligned}$$

We must consider whether such a rest points exists. The rest point $\tilde{q}_{h}$ exists if and only if it is smaller than 1 which gives:

35 $$\begin{aligned} 0.5W\left( 1-\dfrac{n}{K}\right) <d\left( \left( W_{b}+0.5W\right) \left( 1- \dfrac{n}{K}\right) +b\right) . \end{aligned}$$

meaning that the expected number of newborns surviving to maturity raised by survivors of a Hawk–Hawk fight should be lower than the expected reproductive success lost due to death from injuries.

The nontrivial stable size is positive, when

36 $$\begin{aligned} q_{h}<\sqrt{\dfrac{1}{d}\left( 1-\dfrac{1}{\left( W_{b}+0.5W+b\right) } \right) } \end{aligned}$$

which requires that $(W_{b}+0.5W+b)>1$. Let us examine the above formula for $q_{h}=1$. We would have a positive population size if

37 $$\begin{aligned} (1-d)(W_{b}+0.5W+b)>1 \end{aligned}$$

which is equivalent to the expected reproductive success exceeding 1. Thus if (37) holds, persistence of the population is certain.

Now we will analyze the relationship between the classical approach and our new approach.

### **Theorem 1**

 The classical Hawk–Dove game is equivalent to the new model without density dependence if $W_{b}=0$ and $b=1.$ Then the benefit $G=W$ and cost $C=d(W+2)$. 

(for a proof see Appendix 4).

Thus the benefit is the expected fertility $W$. It is interesting that cost is a function of benefit, which is an effect of the application of pre reproduction mortality. Now we can express abstract costs and benefits from the classical Hawk–Dove game in empirically measurable demographic parameters. For example consider the simplified case without density dependence and with $W_{b}=0, W=2$ and $b=1$. Then inequality (35) collapses to $d>0.5$, which means that a Hawk’s probability of death from a Hawk versus Hawk contest should be $>$0.5. However a lack of details such as density dependence or background mortality supported by our new model suggests that this classical approach cannot be realistic. From the point of view of the new approach, in classical model Doves are immortal, Hawks can die during fight only and population growth is unlimited. The absence of density dependence is especially problematic. In the next section the model in ecological equilibrium will be analyzed and differences between unlimited exponential growth and the more realistic new approach will be shown.

### The system in ecological equilibrium

According to the theory developed in Sect. 3.5 we now consider the situation when the system is in ecological equilibrium, i.e. with population size tracing the stable equilibrium determined by the actual Hawk frequency. This can be done by the substitution of a stable point into the suppression coefficient. Considering (31), (33) and (34) the system reduces to (34) (for the population size) and

38 $$\begin{aligned} \dot{q}_{h}=q_{h}(1-q_{h}) \left(\dfrac{1-b( 1-q_{h}^{2}d)}{(W_{b}+0.5W)(1-q_{h}^{2}d) } (0.5W(1-q_{h}d) -W_{b}q_{h}d)-bq_{h}d\right)\nonumber \\ \end{aligned}$$

Now let us analyze the rest points of this system. These are the intersections of the manifolds described by (33) and (34) However, we will limit analysis to frequencies, leaving eco-evolutionary interactions for the next paper.

#### **Theorem 2**

 For this Hawk–Dove game: there is a unique mixed strategy stable equilibrium if

39 $$\begin{aligned} \sqrt{b\frac{1-b}{d}}-0.5<\dfrac{W_{b}}{W}\quad \text{ and}\quad \frac{W_{b}}{W} >0.5(1-b)\left(\frac{1}{d}-1\right) , \end{aligned}$$

a mixed stable equilibrium and a pure Hawk stable equilibrium if

40 $$\begin{aligned} \sqrt{b\frac{1-b}{d}}-0.5<\dfrac{W_{b}}{W}<0.5(1-b) \left(\frac{1}{d}-1\right)\quad \text{ and}\quad \frac{2W_{b}}{W}+1<2b \end{aligned}$$

and a pure Hawk stable equilibrium otherwise. 

If it exists, the stable mixed equilibrium has the form

$$\begin{aligned} \check{q}_{h}=\dfrac{\left( 2\dfrac{W_{b}}{W}+1\right) d-\sqrt{\left( 2 \dfrac{W_{b}}{W}+1\right) d^{2}-4bd\left( 1-b\right) }}{2bd} \end{aligned}$$

and an invasion barrier for a stable pure Hawk equilibrium [where under (38) $q_{h}$ converges to Hawk if and only if $q_{h}>\hat{q}_{h}$]:

$$\begin{aligned} \hat{q}_{h}=\dfrac{\left( 2\dfrac{W_{b}}{W}+1\right) d+\sqrt{\left( 2\dfrac{ W_{b}}{W}+1\right) d^{2}-4bd\left( 1-b\right) }}{2bd} \end{aligned}$$

(for a proof see Appendix 5).

In the case without background fertility $W_{b}$ the general system has the form

41 $$\begin{aligned} \dot{q}_{h}=q_{h}\left( 1-q_{h}\right) \left( 0.5W\left( 1-\dfrac{n}{K} \right) \left( 1-dq_{h}\right) -bdq_{h}\right) \end{aligned}$$

with the scaling parameter

42 $$\begin{aligned} \dot{n}=n\left((1-q_{h}^{2}d) \left( 0.5W\left( 1-\frac{n}{K} \right) +b\right) -1\right) . \end{aligned}$$

Under assumption of ecological equilibrium parameter $W$ vanishes from equations. In this case the rest points from Theorem 2 become $\check{q}_{h}= \dfrac{d-\sqrt{d^{2}-4bd(1-b) }}{2bd}$ and $\hat{q}_{h}=\dfrac{d+\sqrt{d^{2}-4bd(1-b)}}{2bd}$. For either of these solutions to be biologcially meaningful, they must lie within $(0,1)$. Both solutions lie within this range if conditions $0<4b(1-b)<d$ and $b>0.5$ hold. Note that it is not possible that there is exactly one zero point within (0,1). Following the replicator equation (38), if $\check{q}_{h}$ and $\hat{q}_{h}$ lie in the interval $(0,1)$, then $\hat{q}_{h}$ is stable and pure Hawk is also a stable point with invasion barrier $\check{q}_{h}$. It is also perhaps surprising that density dependence has made the pure Hawk population stable. Therefore in the new model the situation known from the classical approach that there is only one rest point in the interior does not occur without background fertility $W_{b}$. We note that without background mortality $(b=1)$ Doves are immortal. This leads to extinction of Hawks and density reaches $K$ (as in Argasinski and Kozłowski 2008).

### Numerical examples

**Fig. 1:**
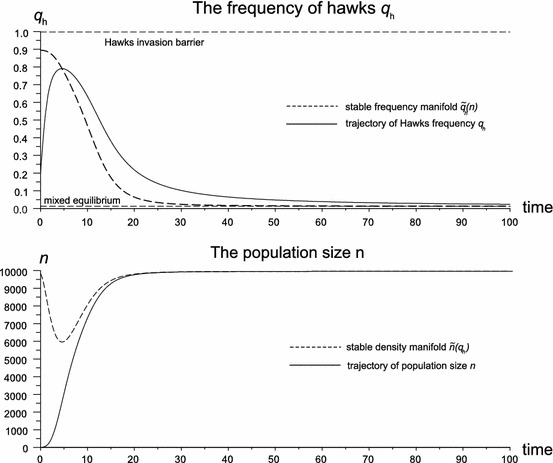
The Hawk frequency increases in low population size but decreases in high population size. This is caused by newborn mortality increasing with population growth, thus making the Hawk strategy not profitable when the population size is large. Parameter values are $W=5,W_{b}=0,d=0.8,b=0.99$ [initial conditions $n(0)=20, q_{h}(0)=0.2]$. The system converges to the mixed equilibrium $\check{q}_{h}=0.0126$, population size $\tilde{n}=9{,}959$, where the Hawk invasion barrier is $\hat{q}_{h}=0.9974$

**Fig. 2:**
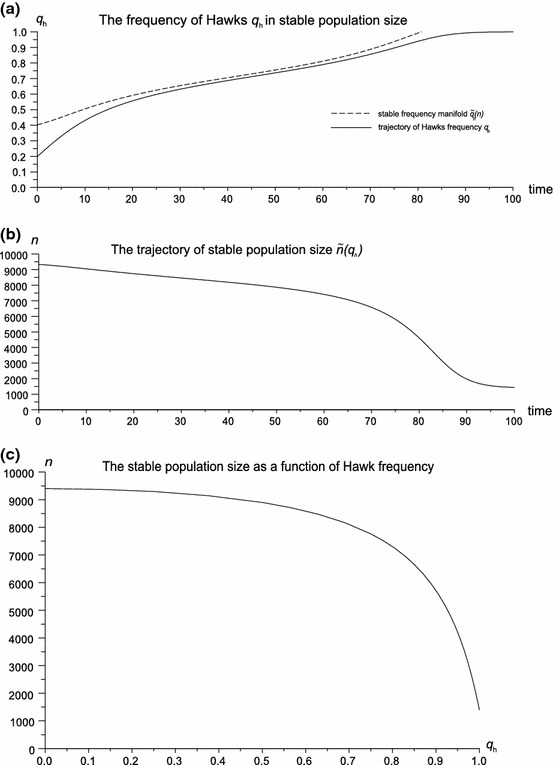
The stable population size is a dynamic equilibrium between mortality and fecundity. An increase of mortality caused by the spreading of Hawks can reduce the population size, even to extinction. **a** A plot of strategy frequencies. The attractor manifold $\tilde{q}_{h}(n)$ exceeds 1, causing the extinction of Doves. **b** The corresponding trajectory of the population size. **c** The stable population size as a function of Hawk frequency. Parameter values are $W=10,W_{b}=0,d=0.8,b=0.7$. In this case there are no mixed equilibria, Hawks outcompete Doves and the population size converges to $\tilde{n}=1{,}400$

**Fig. 3:**
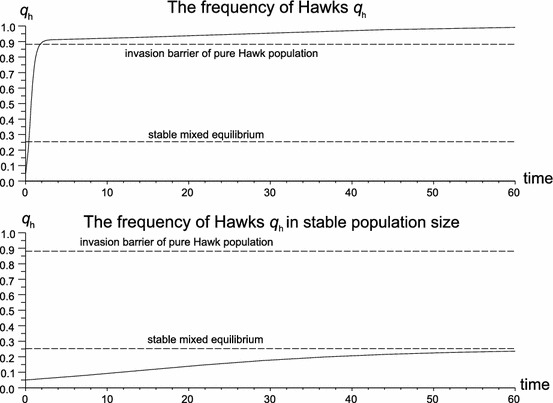
For the same frequency initial conditions $(q_{h}(0)=0.05),$ at low densities (case a, initial population size $n_{0}=20$) Hawks outcompete Doves, when at ecological equilibrium [case b where initial size is determined by (34) and equals $n_{0}=9{,}763$] the system converges to a mixed equilibrium $\check{q}_{h}=0.2616$. Parameters are $W=10, W_{b}=0.1,d=0.5,b=0.9$. The population size converges to $\tilde{n} =7{,}843$. in case a and $\tilde{n}=9{,}734$; in case b, the Hawk invasion barrier is $\hat{q}_{h}=0.8709$

This section contains numerical simulations. Numerical solutions were prepared in two variants: a growing population with initial size $n=20$ and carrying capacity $K=10{,}000$ and a population in ecological equilibrium. The initial Hawk frequency is $q_{h}(0)=0.2$ for Figs. 1 and 2, $q_{h}(0)=0.05$ for Fig. 3. Our new approach is less abstract, and can be used to develop more detailed models. For example, the new model can be interpreted as a mating conflict where individuals compete for females and strategy genes are sex linked and inherited by males. In this case all reproductive success is an outcome of a game, therefore there is no background fertility $W_{b}$ as in the system (41, 42). In this case at low density it is profitable to be a Hawk because of the high payoff from the number of surviving juveniles, however an increase of the population size increases juvenile mortality described by logistic suppression, and in effect the payoff decreases. Thus at high density the Hawk frequency stops growing and begins to decline (Fig. 1).

The model can be extended to the case when individuals are foraging for some resource and when two individuals find a resource then conflict begins. Then the probability of conflict will depend on the density and the availability of the resource. In this case the number of free resources can be described by the background fertility $W_{b}$ and should decrease as the population grows, while the number of resources that can be obtained by conflict $(W)$ should increase. In this case Hawks will decrease in low densities and increase in high ones. This case is worthy of rigorous analysis in subsequent research.

In the preceding paragraph only the frequency dynamics was analyzed in detail. However, the model produces another interesting prediction on density dependence. At ecological equilibrium the frequency dynamics can affect the population size, for example the spread of Hawks can reduce the population size (even to extinction); this is shown in Fig. 2b.

The relationship described by (34) is plotted in Fig. 2c. It shows why the Hawk population becomes fixed in ecological equilibrium. Simply, with the increase of the Hawk frequency, the population size decreases which causes an increase in newborn survivability. Therefore a reduction of the population size makes the Hawk strategy profitable again. This mechanism is shown in Fig. 3. For the same frequency and initial conditions, at low densities Hawks outcompete Doves, whilst at ecological equilibrium the system converges to a mixed equilibrium. This example shows how sensitive the game theoretic structure is to ecological mechanisms such as density dependence.

## Summary of results

### Game theoretic results

In our new approach the game theoretic structure splits into two stages: the mortality and fertility stages. The equilibrium is an effect of the interplay between both stages.

Background fitness can be expressed as background mortality and fertility and it does not vanish from the replicator equations but appears as a multiplicative factor of the mortality stage which has the form $(1-\frac{n}{K})W_{b}+b$.

When the population reaches a stable size manifold consisting of equilibria of the size equation (a type of stationary density surface; Cressman and Garay 2003), conditional on actual strategy frequencies, then the game theoretic structure may change. “Benefit” is affected by density dependent juvenile mortality described by the modifier (23), and in effect the respoint becomes an attractor manifold. Thus, global stationary points are intersections of size and frequency manifolds, and this allows a population of Hawks to become stable. This mechanism is induced by the decrease of population size caused by increased average mortality, which causes a decrease of newborn mortality. The invasion barrier is the Hawk frequency that when surpassed makes the number of offspring surviving to maturity high enough to make the Hawk strategy profitable. Therefore the value of the “benefit” can be modified by neutral density dependence which can in effect seriously change the rules of the game.

This new approach allows us to explicitly describe the cause and effect structure of the modelled phenomenon. The classical theory is phenomenological at the level of determining payoff values expressed in terms of excess over the average growth rate. There are no rules describing how the value of deviation from the average growth rate is determined by strategy. We can always pose the question: why is it not greater (or lower)? In our new approach it can be determined by mortality risk (the probability of death during an interaction with an opponent) and the number of successful reproduction events (for example successful matings). Therefore our new approach can be called “event based modelling”.

### Ecological results

Modern ecological modelling uses very sophisticated mathematical tools such as Lyapunov exponents (Metz et al. 1992). This may cause problems with the understanding of the results obtained by readers with lesser mathematical skills. The framework presented in this paper is an attempt at a solution of this problem, by providing a simple interpretation of parameters. Empirical testing of models formulated under the new framework will be much easier because mortalities (fractions of dead individuals) and fertilities (per capita number of newborns) can be easily measured unlike abstract “costs” and “benefits”. Also costs and benefits are not separated quantities. The Hawk–Dove example shows that “cost” is a function of expected “benefit” defined as a number of offspring. In effect, the general but abstract condition that “cost” should exceed “benefit” collapses to specific but clear and empirically testable statements such as: “if the expected number of offspring is 2 than over half of Hawks should die during a fight for there to be a mixed stable equilibrium”.

Density dependence may affect the structure of the game by increasing newborn mortality; in effect some strategies can be profitable in low densities but not profitable in high densities. The opposite situation can be obtained by density dependent background fertility. Therefore, costs and benefits are not constants. The assumption of constant payoff coefficients was criticized by Dieckmann and Metz (2006) as not realistic. Our model supports their arguments. It shows that even in the application of classical methods, such as logistic suppression, we obtain a varying value of benefit.

The system in ecological equilibrium is not equivalent to the purely frequency dependent case. The equation on frequencies is more complicated, additional rest points may exist (for example in the Hawk–Dove game, a pure Hawk population may become evolutionarily stable). Trajectories of selection determine trajectories of stable population size.

Our new approach shows that the rules of the evolutionary game are not constant but written by ecological conditions and are very sensitive to changes of those conditions. We believe that abstract reasonings related to unspecified “costs” and “benefits” are insufficient. A strong bond with reality via empirically measurable parameters with a clear mechanistic meaning is necessary to produce realistic models. It was clearly shown using the example of the Battle of the Sexes model (Mylius 1999) that more realistic details included in the model (such as realistic pair-formation and the mechanistic interpretation of “costs” as time delays during courtship) can seriously affect predictions, which thus can differ from the “abstract” game model. Therefore, when developing an abstract game theoretic model such a mechanistic analysis should be considered. Our work supports those conclusions.

## Appendix 1: Discrete and continuous models

For a population changing under continuous growth, how do we estimate the parameters from real data? Here we show that we can estimate the real parameters of the model if we record the number of births and deaths within an interval, provided that this interval is sufficiently short, effectively using a straight line approximation to a curve. Thus while we really have a continuously interacting population, we can still get a handle on reality using the method of counting births and deaths as in classical discrete models by comparing our continuous population to a discrete model with a small number of interactions in each time step.

We start by considering the continuous population growth model

43 $$\begin{aligned} \dot{n}_{i}(t)=\tau n_{i}(t)W_{i}-\tau n_{i}(t)d_{i}=\tau n_{i}(t)(W_{i}-d_{i}) \end{aligned}$$

where $n_{i}(t)$ is the number of individuals of the $i$-th type at time $t$. $W_{i}$ and $d_{i}$ are not (necessarily) constants, but can be functions of parameters such as population state or time. The value of $\tau W_{i}$ can be interpreted as the instantaneous deterministic average per-capita birth rate of newborns, $\tau d_{i}$ as the instantaneous deterministic death rate of individuals removed from the population and $\tau$ is the interaction rate (intensity) of individuals.

Thus $W_{i}$ and $d_{i}$ are the value of victory (number of newborns) and the probability of death from a given interaction, respectively. This interpretation, obvious for discrete systems, can be problematic for continuous models, and we justify its use below. Using the zero and first order terms of the Taylor expansion of $n_{i}(t)$ we get

44 $$\begin{aligned} n_{i}(t_{0}+\Delta t)=n_{i}(t_{0})+\tau n_{i}(t_{0})( W_{i}-d_{i}) \Delta t+o(\Delta t). \end{aligned}$$

This should not be confused with a standard discrete population with non-overlapping generations. Rather we imagine a continuously changing population where we count births and deaths within an interval of length $\Delta t$, for successive time periods. In the continuous case interctions are not synchronized and only a fraction $\tau \Delta t$ interacts during $\Delta t$.

For the continuous system to be well-approximated by the first order Taylor expansion, we require the remainder term $o(\Delta t)$ to be small, which occurs for sufficiently small $\Delta t$, when the number of births (respectively deaths) in a time period of length $\Delta t$ is approximated by $\tau n_{i}(t)W_{i}\Delta t$ (respectively $\tau n_{i}(t)d_{i}\Delta t$).

Thus whilst $W_{i}$ and $d_{i}$ are not small in our model, the number of births and deaths within a sufficiently small interval of length $\Delta t$ will be small. By a change of timescale $\tau \Delta t$ can be set to 1 and removed. In effect we obtain equation (5). There is thus a natural link between the number of births (deaths) observed in any time interval and the birth (death) rate from a continuous model and we can use real data on births and deaths to inform the model parameters of our continuous model.

## Appendix 2: Derivation of equations for the Hawk–Dove example game

To evaluate (29) and (30) we need to calculate the following matrix products:

45 $$\begin{aligned} e_{1}S\cdot Pq^{T}&= [1,0]\left[ \begin{array}{cc} 0.5s&1 \\ 0&0.5 \end{array} \right] \left[ \begin{array}{c} q_{h} \\ 1-q_{h} \end{array} \right] =0.5sq_{h}+1-q_{h},\end{aligned}$$

46 $$\begin{aligned} qS\cdot Pq^{T}&= [q_{h},1-q_{h}]\left[ \begin{array}{cc} 0.5s&1 \\ 0&0.5 \end{array} \right] \left[ \begin{array}{c} q_{h} \\ 1-q_{h} \end{array} \right]\nonumber \\&= q_{h}(0.5sq_{h}+1-q_{h}) +0.5(1-q_{h})^{2}. \end{aligned}$$

Thus

47 $$\begin{aligned}&(e_{1}S\cdot Pq^{T}-qS\cdot Pq^{T})\nonumber \\&\quad =(0.5sq_{h}+1-q_{h}) -q_{h}(0.5sq_{h}+1-q_{h})-0.5(1-q_{h})^{2}\end{aligned}$$

48 $$\begin{aligned}&\quad =0.5(1-q_{h})(q_{h}(s-1)+1).\end{aligned}$$

49 $$\begin{aligned}&e_{1}Sq^{T}=[1,0]\left[ \begin{array}{cc} s&1 \\ 1&1 \end{array} \right] \left[ \begin{array}{c} q_h \\ 1-q_h \end{array} \right] =sq_{h}+1-q_{h}=q_{h}(s-1)+1,\end{aligned}$$

50 $$\begin{aligned}&qSq^{T}=[q_{h},1-q_{h}]\left[ \begin{array}{cc} s&1 \\ 1&1 \end{array} \right] \left[ \begin{array}{c} q_h \\ 1-q_h \end{array} \right] =q_{h}(q_{h}(s-1)+1)+(1-q_{h}).\qquad \end{aligned}$$

Thus

51 $$\begin{aligned} (e_{1}Sq^{T}-qSq^{T})&= (q_{h}(s-1)+1) -q_{h}( q_{h}(s-1)+1)-(1-q_{h})\nonumber \\&= (1-q_{h})q_{h}(s-1). \end{aligned}$$

After substitution of the matrix equations (47) and (51) into (29) we get

52 $$\begin{aligned} \dot{q}_{h}&= q_{h}\left( \left( 1-\dfrac{n}{K}\right) (W_{b}( 1-q_{h}) q_{h}(s-1)+W0.5(1-q_{h})(q_{h}(s-1)+1)\right.\nonumber \\&\quad \left.+\,b(1-q_{h}) q_{h}(s-1)\right) \Rightarrow \end{aligned}$$

53 $$\begin{aligned} \dot{q}_{h}&= q_{h}(1-q_{h})\left( \left( 1-\dfrac{n}{K}\right) (0.5W(1-q_{h}(1-s))-W_{b}q_{h}(1-s))-bq_{h}(1-s)\!\right).\nonumber \\ \end{aligned}$$

To complete the system, an equation on the scaling parameter (30) should be added. Since (50) collapses to $q_h^2(s+1)+1$ and (46) collapses to (50) multiplied by 0.5, we obtain:

54 $$\begin{aligned} \dot{n}&= n\left((q_{h}^{2}(s-1)+1)(W_{b}+0.5W) \left(1\!-\!\frac{n}{K}\right) +(q_{h}^{2}(s-1)+1) b-1\right) \Rightarrow \end{aligned}$$

55 $$\begin{aligned} \dot{n}&= n\left( (1-q_{h}^{2}(1-s)) \left((W_{b}+0.5W) \left( 1-\frac{n}{K}\right) +b\right) -1\right). \end{aligned}$$

After the auxiliary substitution $d=1-s$ we obtain equations (31)

56 $$\begin{aligned} \dot{q}_{h}&= q_{h}(1-q_{h}) \left( \left( 1-\dfrac{n}{K}\right) (0.5W(1-q_{h}d)-W_{b}q_{h}d)-bq_{h}d\right),\end{aligned}$$

57 $$\begin{aligned} \dot{n}&= n\left((1-q_{h}^{2}d)\left((W_{b}+0.5W) \left( 1-\frac{n}{K}\right) +b\right) -1\right) . \end{aligned}$$

## Appendix 3: Calculation and stability of the rest points in the Hawk–Dove example game

Let us find the nontrivial rest point. This occurs when

58 $$\begin{aligned}&\left(1-\dfrac{n}{K}\right) (0.5W(1-q_{h}d) -W_{b}q_{h}d)-bq_{h}d=0\Rightarrow \end{aligned}$$

59 $$\begin{aligned}&0.5W\left( 1-\dfrac{n}{K}\right) -q_{h}d\left((W_{b}+0.5W) \left( 1-\dfrac{n}{K}\right) +b\right) =0\Rightarrow \end{aligned}$$

60 $$\begin{aligned}&\tilde{q}_{h}=\dfrac{0.5W\left( 1-\dfrac{n}{K}\right) }{d\left( (W_{b}+0.5W) \left( 1-\dfrac{n}{K}\right) +b\right)}. \end{aligned}$$

This rest point exists if and only if $\tilde{q}_{h}<1$, i.e.

61 $$\begin{aligned} 0.5W\left( 1-\dfrac{n}{K}\right) <d\left((W_{b}+0.5W) \left( 1- \dfrac{n}{K}\right) +b\right). \end{aligned}$$

We find a stable population size if either $\tilde{n}=0$ or

62 $$\begin{aligned} (W_{b}+0.5W) \left( 1-\frac{n}{K}\right) =\dfrac{1-b(1-q_{h}^{2}d) }{1-q_{h}^{2}d} \end{aligned}$$

which in turn gives

63 $$\begin{aligned} \tilde{n}=K\left( 1-\dfrac{1-b(1-q_{h}^{2}d) }{( W_{b}+0.5W)(1-q_{h}^{2}d)}\right). \end{aligned}$$

Stability of (63) comes from the form of the Eq. (32 ). In both of the cases (60) and (63) coefficients describing these nontrivial rest points are negatively linear. This implies stability, because for lower values growth is positive and for greater values it is negative. This nontrivial stable size should be non-negative, so that the population can persist, when

64 $$\begin{aligned}&1-\dfrac{1-b(1-q_{h}^{2}d)}{(W_{b}+0.5W) (1-q_{h}^{2}d)}>0\Rightarrow \end{aligned}$$

65 $$\begin{aligned}&\frac{W_{b}+0.5W}{W_{b}+0.5W+b}<(W_{b}+0.5W)(1-q_{h}^{2}d)\Rightarrow \end{aligned}$$

66 $$\begin{aligned}&1-q_{h}^{2}d>\frac{1}{W_{b}+0.5W+b}\Rightarrow \end{aligned}$$

67 $$\begin{aligned}&q_{h}<\sqrt{\dfrac{1}{d}\left( 1-\dfrac{1}{(W_{b}+0.5W+b) } \right) }. \end{aligned}$$

Thus it is clear that $(W_{b}+0.5W+b)>1$. To consider the possibility of extinction, let us examine inequality (67) for $q_{h}=1$, when the mortality pressure is at its greatest. If a non trivial stable population size exists even in this case, then extinction cannot occur. Here we need

68 $$\begin{aligned} \sqrt{\dfrac{1}{d}\left( 1-\dfrac{1}{(W_{b}+0.5W+b) }\right) }>1 \end{aligned}$$

to be satisfied. Rearranging (68) gives

69 $$\begin{aligned}&1-\dfrac{1}{(W_{b}+0.5W+b)}>d\Rightarrow \end{aligned}$$

70 $$\begin{aligned}&1-d>\frac{1}{(W_{b}+0.5W+b)}. \end{aligned}$$

## Appendix 4: Proof of Theorem 1

To compare both approaches we should remove from the new model factors not considered in the classical approach. In the Maynard Smith model there is no density dependence and no explicit analysis of background fitness. Therefore we should assume that background survivability $b$ is equal to 1, background fertility $W_{b}$ is 0 and there is no suppression coefficient.

The classical matrix has the form

$$\begin{aligned} T=\left( \begin{array}{c|cc}&H&D \\ \hline H&0.5(G-C)&G \\ D&0&0.5G \end{array} \right). \end{aligned}$$

In the new model the equivalent matrix can be presented in the form $e_{1}(S\cdot F+S)q^{T}$ which after the substitution $d=1-s$, is equal to

71 $$\begin{aligned}&\left( \begin{array}{cc} 0.5W(1-d)&W \\ 0&0.5W \end{array} \right) +\left( \begin{array}{cc} 1-d&1 \\ 1&1 \end{array} \right) \nonumber \\&\quad =\left( \begin{array}{cc} 0.5W(1-d)&W \\ 0&0.5W \end{array} \right) -\left( \begin{array}{cc} -d&0 \\ 0&0 \end{array} \right) +\left( \begin{array}{cc} 1&1 \\ 1&1 \end{array} \right) , \end{aligned}$$

where the last matrix can be neglected as a background fitness, therefore we obtain the matrix which entries can be compared with entries of $T$

$$\begin{aligned} \left( \begin{array}{c|cc}&H&D \\ \hline H&0.5W(1-d) -d&W \\ D&0&0.5W \end{array} \right). \end{aligned}$$

Therefore the benefit $G$ is the fertility, i.e. $G=W$ and the cost can be expressed by:

72 $$\begin{aligned}&0.5G-0.5C=0.5W(1-d)-d\Rightarrow \end{aligned}$$

73 $$\begin{aligned}&C=W-W(1-d)+2d=d(W+2). \end{aligned}$$

As we can see cost is a function of benefit. $C>G$, so that there is a mixed stable equilibrium, when

74 $$\begin{aligned} d(W+2)>W, \end{aligned}$$

given by $q_{h}=\dfrac{G}{C}=\dfrac{W}{d(W+2)}.$ We obtain exactly the same formula for the new model equilibrium $\tilde{q}_{h}$ without density dependence, background fertility $(W_{b}=0)$ and mortality $(b=1).\square$

## Appendix 5: Proof of Theorem 2

From (34) we have

75 $$\begin{aligned} \left( 1-\dfrac{\tilde{n}}{K}\right) =\dfrac{1-b( 1-q_{h}^{2}d)}{(W_{b}+0.5W)(1-q_{h}^{2}d)}. \end{aligned}$$

This is the value of the logistic suppression coefficient after the substitution of the stable population size $\tilde{n}$. After substitution of this formula into the equation for the rest point $\tilde{q}_{h}$ (33) we obtain the following formula for the stable Hawk frequency in the ecological equilibrium:

76 $$\begin{aligned} q_{h}&= \dfrac{0.5W\dfrac{1-b(1-q_{h}^{2}d) }{( W_{b}+0.5W)(1-q_{h}^{2}d)}}{d\left((W_{b}+0.5W) \dfrac{1-b(1-q_{h}^{2}d) }{( W_{b}+0.5W) (1-q_{h}^{2}d)}+b\right) } \nonumber \\&= 0.5W\dfrac{1-b(1-q_{h}^{2}d)}{d(W_{b}+0.5W)}= \dfrac{bdq_{h}^{2}+1-b}{d\left( \dfrac{2W_{b}}{W}+1\right)}. \end{aligned}$$

After the substitution $L=\dfrac{2W_{b}}{W}+1$ we obtain the formula $q_{h}= \dfrac{bdq_{h}^{2}+1-b}{dL}$ that leads to the quadratic equation

77 $$\begin{aligned} f(q)=bdq_{h}^{2}-dLq_{h}+1-b=0. \end{aligned}$$

The coefficient of $q^{2}$ in $f(q)$ is positive, so that $f(q)$ has a single maximum as a turning point.

### *Case 1*

Defining $$\Delta =(dL)^{2}-4b(1-b)d=(dL)^{2}-4b(1-b)d$$, if

78 $$\begin{aligned} \Delta <0\Rightarrow L<2\sqrt{\frac{b(1-b)}{d}} \end{aligned}$$

then there are no roots to (77) and it is easy to see that this yields pure Hawk as the unique stable equilibrium. Existence of a single rest point in the interior can be realized by the condition $\Delta =0$. This is an example of an unrealistic (non-generic) solution due to a precise coincidence of ecological parameters.

Otherwise for $\Delta >0$ there are two roots given by

79 $$\begin{aligned} \check{q}_{h}=\dfrac{dL-\sqrt{\Delta }}{2bd}=\dfrac{Ld-\sqrt{ L^{2}d^{2}-4bd(1-b)}}{2bd} \end{aligned}$$

and

80 $$\begin{aligned} \hat{q}_{h}=\dfrac{dL+\sqrt{\Delta }}{2bd}=\dfrac{Ld+\sqrt{ L^{2}d^{2}-4bd(1-b) }}{2bd}. \end{aligned}$$

Since $\Delta <(dL)^{2}$, it is clear from (79) that if $\Delta >0$ then $0< \check{q}_{h}<\hat{q}_{h}$, which gives three possible cases when (78) does not hold.

### *Case 2*

$\check{q}_{h}<\hat{q}_{h}<1$. In this case there is a stable root and so a mixed stable equilibrium at $\check{q}_{h}$, and a pure Hawk stable equilibrium, with invasion barrier at the unstable root $\hat{q} _{h}$. This occurs if (78) does not hold and the following condition (81) holds

81 $$\begin{aligned} dL+\sqrt{\Delta }<2bd \Rightarrow (2b-L)d>\sqrt{\Delta }, \end{aligned}$$

where (81) follows from the condition $\hat{q}_{h}<1$. (81) implies that

$$\begin{aligned} 2b>L, \end{aligned}$$

as well as $(2bd-Ld)^{2}>\Delta$ which leads to $4b^{2}d^{2}-4bd^{2}L+(dL)^{2}>(dL)^{2}-4b(1-b)d \Rightarrow$

$$\begin{aligned} L<b+\dfrac{(1-b)}{d}. \end{aligned}$$

### *Case 3*

$\check{q}_{h}<1<\hat{q}_{h}$. In this case there is a stable root, which is the unique mixed stable equilibrium, at $\check{q}_{h}$. This occurs if (78) does not hold, and the following condition (82) holds we obtain

82 $$\begin{aligned} (L-2b)d<\sqrt{\Delta }<(2b-L)d. \end{aligned}$$

where (82) follows from the conditions $\check{q}_{h}<1$ and $\hat{q}_{h}>1$. The conditions in (82) are equivalent to $\Delta >(2bd-dL)^{2}$ which implies that

$$\begin{aligned} L>b+\dfrac{(1-b)}{d}. \end{aligned}$$

### *Case 4*

$1<\check{q}_{h}<\hat{q}_{h}$. In this case there are no roots in the allowed region and, as when (78) holds, there is a pure Hawk stable equilibrium. This occurs if (78) does not and $\check{q}_{h}>1$ which leads to

83 $$\begin{aligned} (L-2b)d>\sqrt{\Delta }. \end{aligned}$$

(83) implies that $L<2\sqrt{b\frac{1-b}{d}}$ (i.e. $\Delta <0$) or

$$\begin{aligned} 2b<L, \end{aligned}$$

(the opposite to that in Case 2) and

$$\begin{aligned} L<b+\dfrac{(1-b)}{d}. \end{aligned}$$

Thus in summary we have a unique mixed stable equilibrium if

84 $$\begin{aligned} 2\sqrt{b\frac{1-b}{d}}<L\quad \text{ and}\quad L>b+\frac{1-b}{d}, \end{aligned}$$

or equivalently

85 $$\begin{aligned} \sqrt{b\frac{1-b}{d}}-0.5<\dfrac{W_{b}}{W}\qquad \text{ and}\qquad \frac{W_{b}}{W} >0.5(1-b)\left(\frac{1}{d}-1\right); \end{aligned}$$

there is a mixed stable equilibrium and a pure Hawk if

86 $$\begin{aligned} 2\sqrt{b\frac{1-b}{d}}<L<b+\frac{1-b}{d}\quad \text{ and}\quad L<2b \end{aligned}$$

or equivalently

87 $$\begin{aligned} \sqrt{b\frac{1-b}{d}}-0.5<\dfrac{W_{b}}{W}<0.5(1-b) \left(\frac{ 1}{d}-1\right)\quad \text{ and}\quad L<2b; \end{aligned}$$

and there is a pure Hawk stable equilibrium otherwise.

In the special case where $W_{b}=0,L=1<b+(1-b)/d$ so there cannot be a unique mixed stable equilibrium, and so that there is always a pure Hawk stable equilibrium. Using (86) we obtain a mixed stable equilibrium as well if $b>0.5$ and $d>4b(1-b)$. $\square$
